# Effects of Er,Cr:YSGG Laser Surface Treatments and Composites with Different Viscosities on the Repair Bond Strength of CAD/CAM Resin Nanoceramic

**DOI:** 10.3390/polym16152212

**Published:** 2024-08-02

**Authors:** Alperen Degirmenci, Beyza Unalan Degirmenci

**Affiliations:** 1 Department of Restorative Dentistry, University of Van Yuzuncu Yil, 65090 Van, Turkey; 2 Department of Prosthodontics, University of Van Yuzuncu Yil, 65090 Van, Turkey; beyzaunalan@yyu.edu.tr

**Keywords:** micro-shear bond strength, CAD/CAM, repair, composite resin, nanoceramic resin, surface conditioning, Er,Cr:YSGG laser

## Abstract

This study aims to evaluate the repair micro-shear bond strength of the CAD/CAM resin nanoceramic block treated using four different surface treatments and composite resins of different viscosities. For the current study, 96 samples with dimensions of 14 × 12 × 2 mm were obtained from a CAD/CAM resin nanoceramic block (Cerasmart) with a low-speed precision cutting saw under water cooling. The relevant samples were randomly divided into four groups according to the surface treatment processes: grinding with diamond bur, aluminum oxide airborne-particle abrasion, long-pulse laser, and short-pulse laser. Following silane application, universal adhesive was applied to all surface-treated samples and cured with an LED for 10 s. The samples prepared for the repair procedure were divided into two subgroups (microhybrid composite and injectable composite) according to the viscosity of the repair material to be used (*n* = 12). After the repair procedure, care was taken to keep the samples in distilled water in an incubator at 37 °C for 24 h. The repair micro-shear bond strength values (μSBSs) of CAD/CAM resin nanoceramic-composite resin complexes were tested. In addition, randomly selected samples from each group were examined with a scanning electron microscope to evaluate the surface topography after both surface treatments and the micro-shear bond strength test. Data were analyzed by two-way ANOVA and Bonferroni test. It was determined that the surface treatment preferred in the repair protocol significantly affected the μSBS value (*p* < 0.001). While the highest μSBS value was obtained with the short-pulse laser airradiation group, the lowest μSBS values were found in samples with long pulse laser irradiation. However, samples grinded with a bur and airborne-particle abrasion showed similar μSBS values (*p* > 0.05). The preferred composite viscosity in the repair procedure has a significant effect on the μSBS value (*p* < 0.001). However, the interaction between the surface treatment and the viscosity of the repair composite does not affect the μSBS values in a statistically significant way (*p* = 0.193). It may be recommended to clinicians to repair CAD/CAM resin nanoceramic restoration surfaces with injectable composites or after treatment with short-pulse lasers.

## 1. Introduction

Computer-aided design and computer-aided manufacturing (CAD/CAM) systems have unequivocally been among the most rapidly advancing technologies in the dental industry since their introduction in 1985 [1]. This rapid progress led to a significant transformation in several areas of dentistry, particularly prosthodontics and restorative dentistry, and was succeeded by the incorporation of technological systems into biomaterial science [2]. Modern chairside CAD/CAM materials, featuring a range of monomer contents including ceramics, polymers, composite resins, and acrylics, serve as viable alternatives to traditional ceramic blocks. These advancements have expanded the options available for dental applications, offering a diverse selection of materials for consideration [3]. The primary goal of the manufacturers in selecting these monomers and their combinations is to harness the benefits of different materials, resulting in a composite material with improved mechanical and optical properties. The intention is to enhance flexural properties and millability, and minimize the abrasive effect to protect the opposing tooth [4]. The synthesis of dispersed filled resin composites and resin nanoceramics with polymer infiltrated ceramic networks has facilitated the production of several notable products, such as Vita Enamic, Paradigm MZ100, Lava Ultimate, Brilliant Crios, Numerys HC, Grandio Block, Tetric CAD, and Cerasmart. These resin nanoceramic materials, characterized by diverse brands and functionalities, are presently available for purchase [5].

Cerasmart is a high-density nanohybrid structure comprising 71% silica particles and a 29% polymer matrix of bisphenol-A ethoxylate dimethacrylate (Bis-MEPP), urethane dimethacrylate (UDMA), and dimethacrylate (DMA). This resin nanoceramic is characterized by its flexibility and force-absorbing properties. It can be produced in a single session without the need for glazing or sintering [4]. In the study conducted by Suksuphan et al. to assess the fracture resistance of CAD/CAM hybrid dental crown materials, it was underscored that Cerasmart exhibits the ability to endure a loading force surpassing 2000 N without displaying any visible crack formation, even when the occlusal thickness is as minimal as 0.8 mm [6]. The aforementioned hypothesis has been replicated by several researchers, with consistent results being reported [7,8]. However, Yli-Urpo et al. observed that the load-bearing capacity of lithium disilicate glass ceramics surpassed that of Cerasmart in occlusal veneer restorations. They noted that the occurrence of fractures was inevitable, leading to a restricted clinical lifespan of Cerasmart restorations. This phenomenon was attributed to parafunctional movements or internal stress [9].

The literature indicates a consensus that the preferred approach is a repair rather than restoration replacement, especially for fractures in adhesively cemented restorations [3]. According to Hickel et al., the intraoral repair procedure for resin nanoceramics is simplified due to the absence of the need for hydrofluoric acid in the repair process [10]. However, Doğan et al. have highlighted the significant role of surface treatment techniques in the successful repair of resin nano-ceramic restorations. They emphasize the need for a thorough evaluation of the efficacy of current repair procedures [11]. Studies with this focus have introduced the irradiation method using Er:YAG, Nd:YAG, and Er,Cr:YSGG lasers as a clinically acceptable alternative [12,13]. In a recent study assessing the use of laser irradiation for repairing resin nanoceramic restorations, the technique demonstrated effective success. Nevertheless, it has been noted that the specific type of laser and the parameters utilized have a substantial impact on the strength of the repair, and there is currently no consensus on this matter in the literature [14]. Soares et al. have identified a critical gap in the current literature pertaining to the repair of monolithic restorations. Their scrutiny emphasizes the undue focus placed on surface treatment, neglecting the potential impact of the repair composite. This oversight calls for a reevaluation of factors influencing the success of such repairs. The research results unveiled that new generation flowable composites outperformed traditional composites in achieving higher repair bond strength [15].

Upon close examination of the literature, it is evident that the assessment of flowable composites, along with their new reinforced and enhanced iterations, as repair materials, constitutes crucial data. Furthermore, the potential interaction between the existing materials and the newly recommended surface treatment methods remains uncertain. Hence, it is imperative to address these issues through comprehensive research studies, which will provide valuable guidance for enhancing clinical practice. Based on knowledge of current researchers, there are no studies that have evaluated the combined effects of new generation injectable composites used as repair composites and Er,Cr:YSGG laser irradiation as surface treatment and compared with traditional procedures in the repair of resin nanoceramics.

The aim of this preliminary in vitro study is to evaluate the repair bond strength (μSBS) of resin nanoceramic CAD/CAM restoration surfaces using composite resins of varying viscosities, along with four different surface treatments. And the null hypotheses tested are: (1) different surface treatments and (2) viscosity of the repair composite have no effect on the μSBS values.

## 2. Materials and Methods

In this research, a single CAD/CAM resin nanoceramic (Cerasmart, GC Dental Products, Leuven, Belgium), a microhybrid composite (G-ænial Anterior, GC Dental Products, Tokyo, Japan), and an injectable composite (G-ænial Universal Injectable, GC Dental Products, Tokyo, Japan) were utilized as detailed in Table 1.

### 2.1. Calculation of Sample Size

The research team used G*Power 3.1 software to determine the necessary sample size prior to commencing the study. Based on the analysis conducted using a normal distribution, a significance level of 0.05, an effect size of 0.5, and a power of 0.95, the determined total sample size was 96.

### 2.2. Preparation of Samples

Resin nanoceramic CAD/CAM blocks were precisely cut to 14 × 12 × 2 mm dimensions using a low-speed cutting machine (IsoMet 1000, Buehler Ltd., Lake Bluff, IL, USA) with the application of water cooling to ensure optimal results. The sample dimensions were validated using a digital caliper (Absolute Digimatic, Mitutoyo, Kawasaki, Japan). Any samples that did not meet the desired size criteria were subsequently replaced. Then, the samples were embedded in acrylic resin within a circular plastic frame and then polished using 600–800–1000 and 1200 grit silicon carbide papers, each for a duration of 60 s with consistent finger pressure.

### 2.3. Surface Treatments and Bonding Procedure

**Figure 1:**
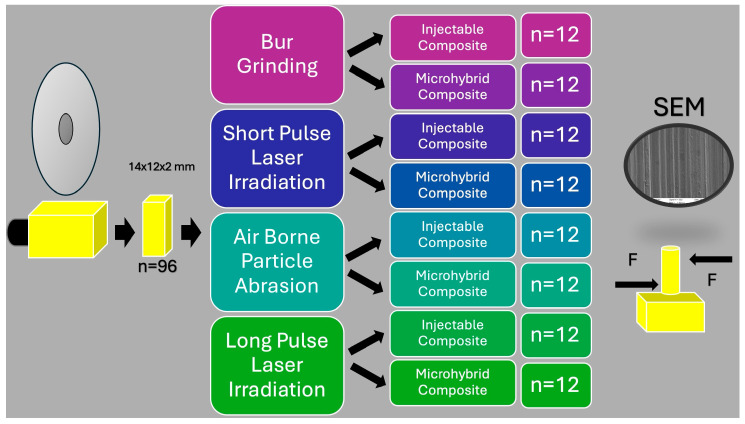
Schematic flowchart of the study.

The samples were randomly divided into four groups based on the intended surface treatment to be applied (*n* = 24) (Figure 1).

The initial procedure involved employing a diamond bur for grinding to accurately replicate the clinical scenario. In order to standardize the procedure, the resin nanoceramic surfaces were initially marked with a permanent marker and subsequently roughened using a 150 μm grit-sized green belt diamond bur (837L-014 GL, Bosphorus, Istanbul, Turkey). The bur was placed parallel to the surface for 4 s with the aid of high-speed rotary tools under water cooling to eliminate the paint trace. Meticulous care was taken to ensure the complete removal and replacement of the burs every five samples [5].

In the second surface treatment, the restoration surfaces undergo aluminum oxide airborne-particle abrasion using 110-micron-sized aluminum oxide (Al_2_O_3_) particles (CoJet Sand, 3M ESPE Co., St. Paul, MN, USA). This process is carried out at a 2.5 bar air pressure from a distance of 10 mm for a duration of 15 s [16].

In the application of the third and fourth surface treatments, the long-pulse and short-pulse laser irradiation protocol involved the use of a 2780 nm wavelength Er,Cr:YSGG laser (Waterlase, Biolase Technology, San Clemente, CA, USA). In the long-pulse laser irradiation protocol, the laser parameters were configured as follows: utilizing the S mode, a pulse duration of 700 μs, a power of 6 W, a frequency of 20 Hz, and a composition of 60% air and 30% water. For the short-pulse irradiation, the parameters were set to H mode, with a pulse duration of 60 μs, a power of 6 W, a frequency of 20 Hz, and a composition of 60% air and 30% water. In both procedures, the surfaces were roughened using a 600 µm diameter fiber tip at a distance of 1 mm for a duration of 20 s. Upon completion of surface treatments, the samples underwent a 5-min cleaning process in an ultrasonic bath, followed by gentle drying with a paper towel. Ceramic Primer II was applied to the surface of the sample in a thin layer and dried with air spray. The application of a universal adhesive (G-Premio Bond) to the surface was carried out using an applicator. Subsequently, the adhesive was allowed to set for 10 s and then dried with maximum air pressure for 5 s. Following this, the samples were exposed to visible blue light with a wavelength of 430–480 nm and a light intensity of 1400 mW/cm^2^ by an LED light device (D-Light Pro, GC Corporation, Tokyo, Japan) at a distance of 10 mm for a duration of 10 s. The LED light device’s light intensity calibration involved the examination of one in five samples with the aid of a radiometer (LED Radiometer, SDI Dental Limited, Melbourne, Australia). Resin nanoceramic samples, within all surface treatment groups, were segmented into two subgroups based on the type of repair composite intended for use: injectable composite and microhybrid composite (*n* = 12). The appropriate repair composite was applied using a mold on the surfaces, ensuring it did not exceed 2 mm in thickness. Both resin composites were cured with the same LED device at a light intensity of 1400 mW/cm^2^ for 10 s, in accordance with ISO 4049 standards [17]. The prepared resin nanoceramic-repair composite complexes were then stored in distilled water in a 37 °C incubator for 24 h before conducting the repair bond strength test.

### 2.4. Repair Bond Strength Test (μSBS)

The repair bond strength test was performed using a micro-shear testing device. The samples were positioned in parallel on the mounting jigs of the testing machine (Bisco Shear Bond Tester, Bisco, Schaumburg, IL, USA), and a shear load was applied at a crosshead speed of 1.0 mm/min until the complex fractured. The data type was determined in megapascals (MPa). The fracture patterns of the specimens were characterized as adhesive, cohesive, or mixed utilizing a light microscope (TM-505, Mitutoyo, Tokyo, Japan).

### 2.5. Scanning Electron Microscope (SEM) Analysis

Two distinct scanning electron microscope (SEM) images were captured from the samples: one following the surface treatment and the other after the μSBS test. Both SEM images were assessed at a magnification of 1500× by two independent researchers.

### 2.6. Statistical Analysis

The suitability of the data for normal distribution was tested with the Shapiro Wilk test. Two-way analysis of variance (ANOVA) and Bonferroni test were used to evaluate μSBS data. Analysis results are presented as mean ± standard deviation. The statistical analysis was conducted using the IBM SPSS software version 23.0, with a significance level set at *p* < 0.050.

## 3. Results

**Figure 2:**
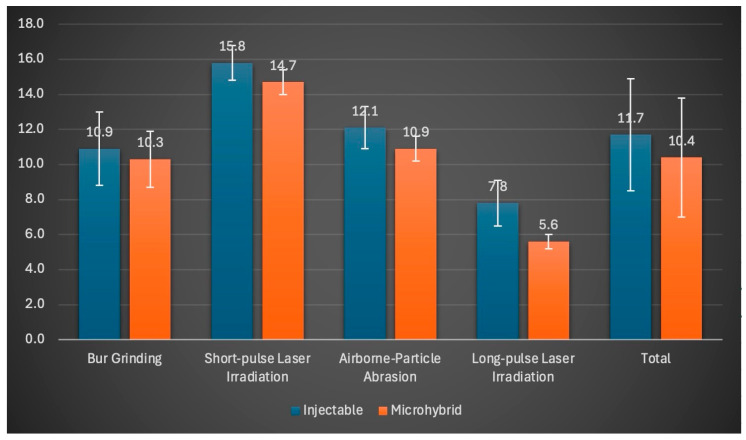
Means and standard deviation data of μSBS values (MPa) of surface treatments.

The mean μSBS values for the repaired CAD/CAM resin nanoceramic specimens are presented in Figure 2.

The results of the two-way analysis of variance indicate a statistically significant influence of the surface treatment applied to the resin nanoceramic prior to repair and the type of composite used during the repair procedure on the μSBS value (*p* < 0.001, F = 193.667; *p* < 0.001, F = 25.284) (Table 2). However, the interaction between surface treatment and repair composite was not found to be statistically significant (*p* = 0.193). The complexes repaired with injectable composite and subjected to short-pulse laser irradiation exhibited the highest μSBS value of 15.8 MPa. In contrast, the samples repaired using the combination of long-pulse laser irradiation and microhybrid composite demonstrated the lowest μSBS value. One of the most noteworthy observations extracted from the data is the markedly elevated repair bond strength values demonstrated by samples repaired with injectable composites, irrespective of the surface treatment applied (*p* < 0.001). The repaired samples utilizing microhybrid composite exhibited a μSBS value limited to 10.4 MPa. Remarkably, the highest repair bond strength of 15.3 MPa was observed subsequent to short-pulse laser irradiation, followed by airborne-particle abrasion, bur grinding, and long-pulse laser irradiation groups, in descending order of efficacy.

Following the μSBS test, Table 3 illustrates the distribution of failure types. It was determined that adhesive failure emerged as the predominant failure type across all groups.

**Figure 3:**
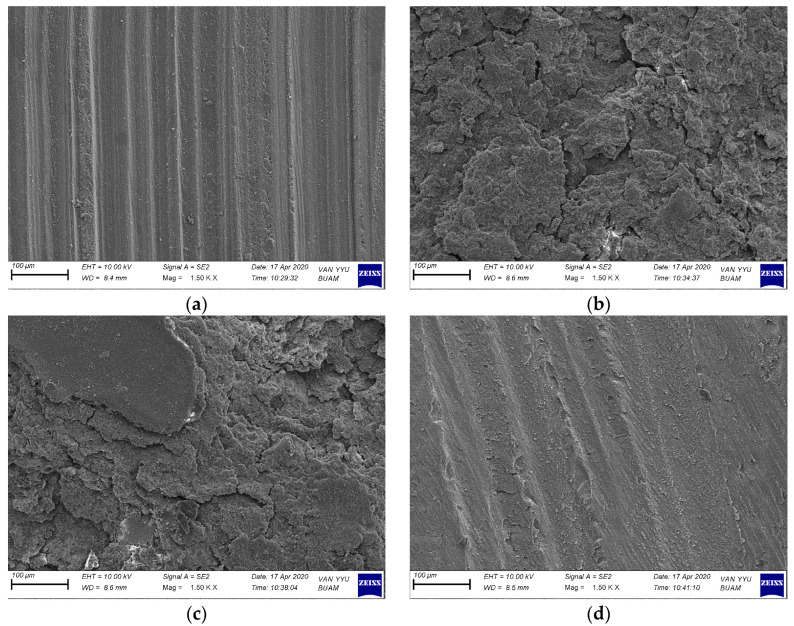
SEM image (1500×) of the surface of the CAD/CAM material after applying the surface treatment. (**a**) After bur grinding; (**b**) after short-pulse laser; (**c**) after long-pulse laser; (**d**) after aluminum oxide airborne-particle abrasion.

The SEM microphotographs displayed in Figure 3 illustrate the surface characteristics of CAD/CAM resin nanoceramics following various treatments. Figure 3a showcases the remarkable formation of distinct, vertical grooves on the surface after bur grinding. In Figure 3b, the surface appears etched following short-pulse laser irradiation, resulting in a foamy appearance, possibly correlating with the observed μSBS value. Figure 3c demonstrates the similar etching and foamy appearance resulting from long-pulse laser irradiation, with noticeable flat areas unaffected in comparison to short-pulse laser treatment. Lastly, Figure 3d exhibits the presence of vertical lines on the surface following airborne-particle abrasion, albeit without the formation of deep grooves as seen with bur grinding.

**Figure 4:**
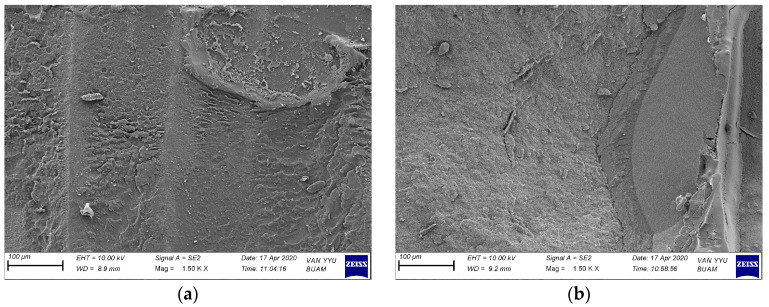

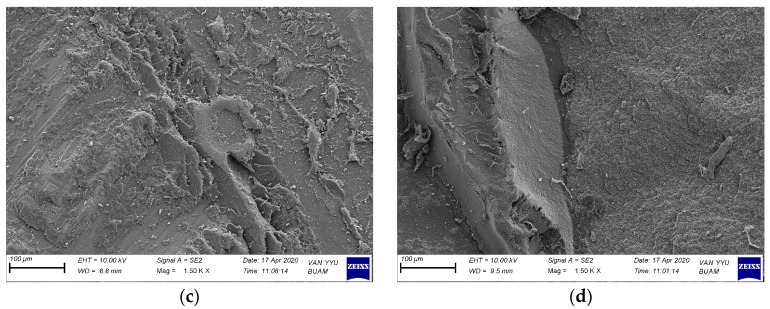
SEM image (1500×) of the surface of the CAD/CAM material after being repaired with composite and then after the SBS test is performed (**a**) after bur grinding; (**b**) after short-pulse laser; (**c**) after airborne-particle abrasion; (**d**) after l2ong-pulse laser.

The SEM microphotographs presented in Figure 4 display randomly selected samples following the μSBS test. The images clearly showcase repair composite residues, indicating adhesive failure subsequent to the relevant surface treatments.

## 4. Discussion

The evaluation of material loss or repairability of small fractures in CAD/CAM resin nanoceramics holds significant clinical relevance in determining the longevity of restorations. The current in vitro study was conducted to investigate this, and the results indicate that employing short-pulse laser irradiation as a surface treatment led to higher repair bond strength in resin nanoceramics samples (*p* < 0.001), thereby rejecting the first null hypothesis. Furthermore, the study results demonstrated that the viscosity of the repair composites had a statistically significant effect on the μSBS values (*p* < 0.001), leading to the rejection of the second null hypothesis.

In the context of clinical repair procedures, establishing a robust bond between the restoration surface and the composite resin is paramount for achieving success. Consensus within the literature underscores the significance of introducing irregularities to the resin nanoceramic surface via diverse surface treatments or chemical interactions [18].

In their study, Elkassaby et al. underscored the significance of appraising the techniques favored by dentists in their everyday clinical practice. They noted that acid application, bur grinding, and airborne-particle abrasion are frequently employed methods [19]. Niiuzuma et al. stated that the application of hydrofluoric acid is very effective in dissolving the glassy network structure of resin nanoceramics; thus, a porous and bonding-friendly surface can be obtained. However, they also emphasized that there are studies that classify hydrofluoric acid as an extremely dangerous chemical and that importance should be given to restricting its intraoral use [20]. Similarly, Didangelao et al. also highlighted the potential side effects that could arise during the intraoral repair procedure. They suggested that using irradiation with the Er,Cr:YSGG laser as a surface treatment should be encouraged to eliminate these possibilities [21]. In a recent investigation pertaining to this matter, it was affirmed that following Er,Cr:YSGG laser irradiation, surfaces displayed irregular and rough characteristics. Nevertheless, assessment indicated that a satisfactory bond strength could be achieved with a minimum application of 3 W [5]. Tokar et al. found that while the original research solely focused on laser power, the implementation of various modes in the laser irradiation process notably enhanced the repair bond strength [22]. Considering all these data, it was decided to use grinding with a bur, air-borne particle abrasion and Er,Cr:YSGG laser irradiation methods as surface treatment in the current study and to evaluate laser irradiation in two different modes, short and long pulse.

One critical aspect of the restoration repair process involves the application of repair composite. Over the years, scholarly literature has thoroughly examined the repair efficacy of new materials with traditional composites [23]. However, Souza et al. first proposed the notion that different composites might influence the repair process. In their study they observed that the viscosity of the repair composite did not affect the strength value of indirect composites. However, it was noted that composite viscosity could impact various restorative materials, including ceramics [24]. Upon evaluation, the repair bond strength of lithium disilicate reinforced ceramic materials with self-adhering flowable composite resins was found to be low regardless of the surface treatment, leading to the decision not to compare the results with composites of different viscosities [25]. Based on the present authors’ knowledge, there is currently no study in the literature that uses injectable composites, also known as highly filled flowable composite, as a repair composite. Therefore, in light of this gap in the literature, the decision was made to utilize injectable composites and microhybrid composites with varying viscosities in the current study.

Various qualitative and quantitative analyses were conducted to assess the impact of surface treatments favored in the repair of CAD/CAM resin nanoceramic restorations on bond strength [18]. The results of the current study demonstrate that the bond strength of the repair can be significantly enhanced through the utilization of a preferred surface treatment. Notably, the group subjected to short-pulse laser irradiation exhibited the highest μSBS value of 15.3 MPa. In a similar vein, Bahadır et al. conducted an assessment of the repair bond capacity of CAD/CAM resin nanoceramics through the application of Er,Cr:YSGG laser irradiation at 3 W. Their data revealed a resulting bond strength of 14.81 MPa [5]. In a recent study assessing the repair protocol involving Er,Cr:YSGG laser irradiation in resin-based CAD/CAM materials, it was found that a bond strength of 15.23 MPa was achieved. Furthermore, it was observed that the strength value increased to 15.67 MPa with an increment in the laser power parameter [21]. In contrast, another study examining the bond strength for repairs using Er,Cr:YSGG laser treatment found that despite increasing the irradiation power, the resulting bond strength value (μSBS) only reached 9.7 MPa, failing to approach the control group. The researchers concluded that this surface treatment method was ineffective [26]. The disparity in our findings is likely attributable to the use of lithium disilicate and zirconium-based CAD/CAM restoration material in the respective study, alongside the constraint of a 2.5 W limit on the applied power parameter. The glassy phase, filler content, and filler load of these materials exhibit notable distinctions from resin nanoceramics. This disparity stands as a significant contributor to alterations in the interaction between the laser light and the material [5]. In this study, it was observed that the parameters selected for Er,Cr:YSGG laser irradiation significantly impacted the repair bond strength. Specifically, the average μSBS achieved with short-pulse laser irradiation was 15.3 MPa, while long-pulse irradiation resulted in a weaker strength of 6.7 MPa. The difference is mainly attributed to variations in pulse duration during the relevant irradiation procedures, despite using the same power value. Consequently, the interaction between the laser light and the material changes. Assuming that more pulses are administered within a specified time during the short-pulse laser irradiation protocol, where the pulse duration decreases, it is anticipated that greater interaction will occur between the laser light and the glassy phase of the CAD/CAM resin nanoceramic. According to Didangelou et al., excessive pulsation leads to high local temperatures and microexplosions on the surface. This means inorganic particles on the restoration surface can be removed more effectively, and micropores that can aid in the repair can be created on the surface [21]. The SEM samples from the current study showed deeper and more microporosity images in the short-pulse laser irradiation group, which supports the claim. This aligns with the results from a study by Tokar et al., where it was reported that higher bond strength was achieved in zirconia surfaces treated with short-pulse laser irradiation compared to the long-pulse irradiation group [22].

Composites with varying viscosities are presently employed in the restoration of fractures in indirect restorations. Clinicians frequently opt for low-viscosity composites due to their capability to effectively penetrate crack surfaces [27]. The viscosity of composites is influenced by the filler amount, matrix formulation, production method, and any changes in these categories could impact the repair bond strength of the material [28]. Limited research exists on the impact of repair composite viscosity. In a study by Kemaloğlu et al., the effects of using a low-viscosity conventional flowable composite and an injectable composite to repair Cerasmart were investigated. The findings indicated that regardless of the surface treatment, injectable composites are considered the preferred choice for repairing resin nanoceramics, resulting in a significant 12.19 MPa increase in repair bond strength [27]. In this similar study, it was observed that the use of the injectable composite resulted in higher μSBS values. This can be attributed to the injectable composite’s low viscosity and high wettability, which led to the creation of a larger free surface formation. Consequently, limited shrinkage at the adhesive interface and reduced shrinkage stresses allowed the injectable composite to better adapt to the restoration surface, leading to a more successful repair procedure. In a separate preliminary study, researchers compared the impact of injectable composites and traditional paste-type composites on repair strength. They observed that the average repair bond strength achieved with paste-type composites was 17.1 MPa, while it was 17.0 MPa with injectable composites. The researchers underscored the effectiveness of injectable composites as repair materials [28]. In the present study, injectable composites proved to be a successful repair material. The bond strength values obtained from the repair process were significantly higher than those obtained with the paste-type microhybrid composite. This disparity may be attributed to the variations in restoration materials utilized in the study and the differing surface treatments employed during the repair procedure.

Numerous researchers assert that scanning electron microscope (SEM) microphotographs play a pivotal role in assessing the efficacy of surface treatments [4,18]. In their investigation, Bahadır and Bayraktar observed that “SEM images of laser-irradiated surfaces exhibit irregularities and characteristics reminiscent of melting”. At the same time, when the bur groups were examined in this study of the researchers, vertical grooves were seen [5]. In the current study, there are similar SEM images in the bur group. In the study by Didangelou et al., intact surfaces were observed in the control group, while changes in morphology occurred in the treated surfaces when examining SEM images. The researchers tested various laser modes and found that groups E (3.5 W, 35 Hz, Mz6(600 μM), 100 mJ, 90 s) and F (4.5 W, 50 Hz, Mz6(600 μM), 90 mJ, 90 s) yielded the most positive results. In the other groups, intact surface islands were formed, potentially hindering adhesion with the repair resin composite, likely due to the pulsed mode of laser irradiation [21]. In our investigation, specifically in the short-pulse laser mode, there is noticeable surface roughness. Conversely, in the long-pulse laser mode, there are undamaged surface islands, consistent with the findings of Didangelou et al. Upon reviewing the μSBS data from the current study, it appears that the long-pulse laser mode exhibits lower performance, potentially due to the presence of intact areas. In the study conducted by Şişmanoğlu et al., it was observed that various air-abrasion techniques led to surface alterations and the presence of irregular particles on the CAD/CAM material surface. Conversely, the application of hydrofluoric acid as a surface treatment induced the formation of notable protrusions and deep pores [29]. In our study, it is observed that less significant wear occurred in the sandblasting process compared to the other groups.

The text discusses the evaluation of failure modes using μSBS data. While most studies have used light microscopy to analyze these modes, some have also utilized SEM data. A recent repair study suggests that there is no clear link between failure modes, aging, and surface treatment, indicating that failures are somewhat random. It is important to note that the number of adhesive failures increases in all groups after thermal cycling, while the number of mixed failures decreases. The laser groups generally exhibit mixed failures, whereas the air-abrasion group has a high rate of cohesive failures. The high rate of cohesive failures in the air-abrasion group is likely due to the increased filler material content, resulting in a more brittle behavior for the tested composite. On the other hand, mixed failures are predominant in the laser groups, indicating that laser modifications can still create a suitable adhesive surface, as demonstrated in other studies [21]. In the study conducted by Kılınç et al., it was found that adhesive errors were predominantly present in the control groups under both aging conditions. Notably, mixed errors were the prevailing observation in the laser groups [13]. In our study, we found that adhesive failure was the predominant type of failure in all groups, with mixed failure also observed in laser groups, aligning with findings from other studies.

The current in vitro study has some limitations due to its nature. Initially, fractures were expected to occur after clinical use; however, the samples were not subjected to any aging procedures. Our evaluation of repair bond strength was confined solely to the micro-shear test. Despite implementing a range of surface treatments and conducting characterization analyses such as FTIR, XRD, and EDS to elucidate the interplay between the surface treatments and the resin nanoceramic surface, these investigations were not carried out. Moreover, we did not factor in the potential impact of saliva contamination during intraoral use on μSBS. It is evident that further in vitro and in vivo studies are essential to comprehensively evaluate all these factors.

## 5. Conclusions

Within these limitations, the following conclusions can be drawn:

1. For the repair of CAD/CAM resin nanoceramic restorations, the most effective approach involves surface irradiation using a short-pulse laser, followed by repair utilizing injectable composite.
2. Regardless of the preferred surface treatment, higher bond strength is achieved when the injectable composite is used as the repair material.

## Figures and Tables

**Table 1:** Materials used in the study.

Material	Type	Composition	Lot Number
Cerasmart	Force absorbing hybrid ceramic CAD/CAM Block	Bis-MEPP ^1^, UDMA ^2^, dimethacrylate 71% silica (20 nm), barium glass (300 nm)	1811221
G-ænial Anterior	Microhybrid composite resin	Matrix (24%): UDMA ^2^, dimethacrylate co-monomers Fillers (76%): pre-polymerized fillers (16–17 μ), strontium glass (400 nm), lanthanoid fluoride (100 nm), silica (16 nm), fumed Silica (16 nm), silica glass	190627A
G-ænial Universal Injectable	Injectable composite resin	Matrix (31%): methacrylate monomer Fillers (69%): silica (16 nm), barium glass (150 nm)	190525A
Ceramic Primer II	Silane	Silane, phosphate monomer, methacrylate, ethanol	2301051
G-Premio Bond	Universal adhesive	4-MET ^3^, phosphate monomer, thiophosphate monomer, dimethacrylate, acetone, water, photoinitiator	2112131

^1^ Bis-MEPP: Bisphenol-A ethoxylate dimethacrylate. ^2^ UDMA: Urethane dimethacrylate. ^3^ 4-MET: 4-methacryloxyethyl trimellitic acid.

**Table 2:** Examination of surface treatment and repair composite effect on μSBS values.

Source	Sum of Squares	Sd	Mean Squares	F	*p*	Partial Eta Squared
Surface treatment	890.661	3	296.887	193.667	<0.001	0.868
Repair composite	38.760	1	38.760	25.284	<0.001	0.223
Surface treatment × Repair composite	7.395	3	2.465	1.608	0.193	0.052

R^2^ = 0.864.

**Table 3:** Distribution of failure modes by groups.

Surface Treatment	Repair Composite	Failure Type		
		Adhesive	Cohesive	Mix
Bur	Injectable	11	1	0
	Microhybrid	11	1	0
Short-pulse laser	Injectable	10	0	2
	Microhybrid	10	0	2
Airborne-particle abrasion	Injectable	12	0	0
	Microhybrid	12	0	0
Long-pulse laser	Injectable	10	1	1
	Microhybrid	10	1	1

## Data Availability

Data are contained within the article.
